# Guiding Documents for Engaging with Remote Chronic Disease Management Programs as a Healthcare Provider: A Scoping Review

**DOI:** 10.5195/ijt.2023.6583

**Published:** 2023-12-12

**Authors:** Jill Van Damme, Vanina Dal Bello-Haas, Ayse Kuspinar, Patricia Strachan, Nicole Peters, Khang Trong Nguyen, Greg Bolger

**Affiliations:** 1 School of Rehabilitation Science, McMaster University, Hamilton, Ontario; 2 School of Nursing, McMaster University, Hamilton, Ontario; 3 School of Rehabilitation Science, Western University, London, Ontario; 4 Durand Health, Hamilton, Ontario

**Keywords:** Chronic disease management, Remote programs, Service delivery, Telerehabilitation, Virtual delivery

## Abstract

**Introduction:**

Chronic disease management programs (CDMP) that include education and exercise enhance outcomes and reduce healthcare costs. Remote CDMP have the potential to provide convenient, cost-effective, and accessible options for individuals, but it is unclear how to best implement programs that include education and exercise. This review identified and synthesized resources for implementing remote CDMP programs that incorporate education and exercise.

**Methods:**

Peer-reviewed and grey literature were systematically searched from January 1998 to May 2022. Covidence software was used for screening and extraction. The data were synthesized and presented in a narrative and tabular format.

**Results:**

Six peer-reviewed manuscripts and six grey literature documents published between 2006–2022 were included. All resources described individual programs targeting various chronic conditions. Provider training, consent, participant screening, and safety considerations were identified.

**Conclusions:**

Guidelines for remote CFMP programs are lacking. Additional work is needed to design remote CDMP guidelines incorporating education and exercise.

Approximately one out of every three individuals worldwide are living with one or more chronic conditions (Hajat & Stein, 2018). Ischemic heart disease, stroke, cancer, depression, diabetes and back and neck pain contribute to higher mortality and morbidity as measured by disability-adjusted life years (Hajat & Stein, 2018). Living with a chronic condition can have negative financial, social, and quality-of-life impacts (Hevey et al., 2018; Hwang et al., 2001), highlighting the importance of effective chronic disease management strategies. It is well established that increasing physical activity is effective in the prevention and management of numerous chronic diseases (Thornton et al., 2016). Similarly, education is also considered a cornerstone of chronic disease management, as people make decisions every day about their diet, lifestyle, medication adherence, and activity that impact their health condition and prognosis (Bodenheimer et al., 2002). Chronic disease management education can include information on the disease itself, development of technical skills (i.e., using a glucose monitor), and self-management skills to improve illness-related problem-solving strategies (Bodenheimer et al., 2002). Interventions that enhance self-management improve individual outcomes and reduce healthcare-related costs (Fortin et al., 2016) owing to greater activation (i.e., developing knowledge, skills, beliefs, and behaviours), self-regulation (i.e., managing thoughts, emotions, and behaviours), and self-efficacy (i.e., personal beliefs about the ability to complete activities) (Moore et al., 2016). There is evidence to support that interventions that combine education and exercise are more effective than those that focus only on exercise or education (Bannuru et al., 2019).

Chronic disease management programs can be delivered in-person (group or individualized) (Borek et al., 2019; Fortin et al., 2013) or remotely via smartphone applications (Antypas & Wangberg, 2014), telephone calls (Allen et al., 2010; Lewis et al., 2017), and videoconferencing (Katz et al., 2018). There are numerous terms used to describe remote chronic disease management programs including telehealth, telemedicine, eHealth, mHealth, virtual care, and telerehabilitation, with differences in the literature regarding how each is conceptualized and implemented. For this review, remote chronic disease management included programs using online or website technologies, including telephone, email, applications, and videoconferencing.

The COVID-19 pandemic has resulted in increased demand for remote healthcare options to ensure that people can access care while maintaining public health safety guidelines (Portnoy et al., 2020). Previous research supports the effectiveness of remote rehabilitation in people with chronic conditions in terms of function, quality of life, satisfaction, and adherence (Seron et al., 2021). Remote formats have demonstrated good patient engagement (i.e., full participation and attendance) (Dal Bello-Haas et al., 2014) and satisfaction with services (i.e., indicated that they would participate in virtual formats again) (Harkey et al., 2020). In addition, remote formats can provide convenient, cost-effective, and accessible care options (Dal Bello-Haas et al., 2014; Harkey et al., 2020; Portnoy et al., 2020). However, there is no clear guidance for healthcare providers on how to best implement a remote program that incorporates both education and exercise to improve the self-management of chronic conditions. The objective of this scoping review is to (1) identify what types of documents exist for clinicians providing remote chronic disease management programs that include both educational and exercise components; and (2) synthesize what recommendations regarding the processes and procedures exist for clinicians implementing a remote chronic disease management program that include both education and exercise.

## Methods

This review followed JBI methodology for scoping reviews (Peters et al., 2020). The *a priori* protocol (Van Damme & Dal Bello-Haas, 2021) is publicly available in accordance with the Preferred Reporting Items for Systematic Reviews and Meta-Analyses Extension for Scoping Reviews (PRISMA-ScR) (Tricco et al., 2018).

This review considered primary research studies, pilot studies, and protocols with quantitative, qualitative, or mixed-method study designs for inclusion. In addition, literature reviews and grey literature, including unpublished manuscripts, opinion papers, commentaries, organizational materials, policies, and guidelines from government sources, non-profit organizations, healthcare providers, and/or regulatory bodies, were also considered for inclusion. Documents from all countries were considered, however only Canadian regulatory bodies for healthcare providers were targeted and searched. Legislative resources were not considered for inclusion.

Inclusion criteria were as follows: (1) adults 18 years of age and older, (2) any sex or gender, (3) discussing a chronic condition, (4) explored or described chronic disease management programs comprising *both* education and exercise, (5) interventions were developed/provided by a licensed and regulated healthcare provider (chiropractors, physiotherapists, registered kinesiologists, registered exercise physiologists, nurses), (6) interventions were remote (i.e., synchronous and asynchronous sessions using mobile applications, web-based technologies, videoconferencing, telephone, gamification, etc., or a combination of formats). Documents that included home visits from healthcare providers were excluded.

An initial limited search of MEDLINE was conducted in consultation with a health sciences librarian to identify key articles, keywords, and index terms. Keywords and index terms were used to develop a full search strategy, which was trialed on June 6, 2021. The search strategy, including all identified keywords and index terms was adapted for CINAHL, EMBASE, AgeLine, PsycINfo, Allied and Complementary Medicine, Global Health, HealthSTAR, and Politics collection, as these databases have a focus on health, medicine, public health, public policy and the health sciences, and health services delivery. The initial search was completed on July 8, 2021, and the updated search was completed on May 9, 2022. The full search strategy for MEDLINE is provided in Table 1 below. The reference lists of the articles and other documents included in the review were screened for additional articles and resources. Only studies published in English were included due to language restrictions of the research team from 1998 to the date of the search, as telehealth was not introduced in Ontario until this time (Williams, 2012).

**Table 1.** MEDLINE Search Strategy Completed July 8, 2021

Search	Query	Records retrieved
#1	(Virtual or telerehabilitation or telehealth or mHealth or eHealth or remote or video^*^).mp. [mp=title, abstract, original title, name of substance word, subject heading word, floating sub-heading word, keyword heading word, organism supplementary concept word, protocol supplementary concept word, rare disease supplementary concept word, unique identifier, synonyms]	345042
#2	(Chronic disease^*^ or chronic condition^*^ or multimorbidity or multiple chronic conditions).mp. [mp=title, abstract, original title, name of substance word, subject heading word, floating sub-heading word, keyword heading word, organism supplementary concept word, protocol supplementary concept word, rare disease supplementary concept word, unique identifier, synonyms]	340905
#3	(manage^*^ or self-manage^*^ or support or program or intervention^*^).mp. [mp=title, abstract, original title, name of substance word, subject heading word, floating sub-heading word, keyword heading word, organism supplementary concept word, protocol supplementary concept word, rare disease supplementary concept word, unique identifier, synonyms]	12126007
#4	(guideline^*^ or recommend^*^ or policy or regulation^*^ or principle^*^).mp. [mp=title, abstract, original title, name of substance word, subject heading word, floating sub-heading word, keyword heading word, organism supplementary concept word, protocol supplementary concept word, rare disease supplementary concept word, unique identifier, synonyms]	3106696
#5	#1 AND #2 AND #3 AND #4 ^*^limited to English	642

All identified studies were uploaded into the Covidence software (www.covidence.org), and grey literature was charted into an Excel spreadsheet. Duplicates were removed and a title-abstract pilot test of 10 journal articles and 10 grey literature documents was completed to calibrate the screening process with the eligibility criteria. The remaining journal articles and grey literature sources were screened by two independent reviewers using Covidence and Excel. Full-text studies that did not meet the inclusion criteria were excluded. Disagreements between the reviewers were resolved through discussion until consensus was reached (journal articles, n=9; grey literature, n=27).

Data were extracted by two independent reviewers in Covidence (Peters et al., 2020). The data extracted included (1) specific details about the target chronic disease, (2) specific format of the chronic disease management program, (3) education details, (4) exercise session details, (5) guidelines for virtual delivery, (6) technology used, (7) healthcare provider-delivering program, (8) geographic location, (9) participant characteristics, and (10) any other key findings relevant to review questions.

## Results

**Figure 1.**
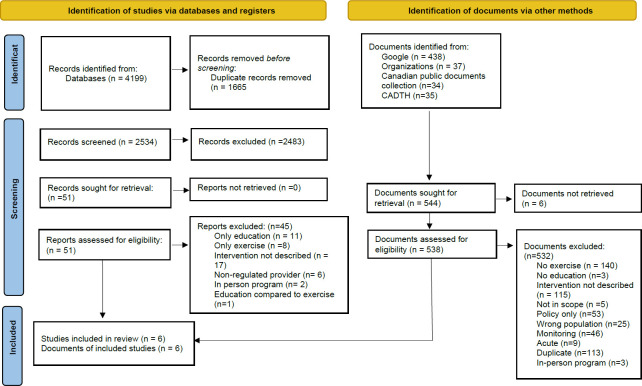
PRISMA 2020 Flow Chart

The search strategy identified 4199 journal articles via the databases searched and 544 documents using other search methods. Forty-seven journal articles and 538 grey literature documents underwent full-text review. Six journal articles published between 2015–2021 were included from database sources, including one protocol, two pilot studies, two randomized control trials, and one expert consensus review article. Six documents published between 2006–2022 were obtained through other search sources, including one commentary journal article, four guideline documents, and clinical guidelines and technical standards. The PRISMA 2020 flowchart (Figure 1) depicts each stage of the systematic search process.

All the identified studies and documents focused on individual remote chronic disease management (i.e., one-on-one, no group settings) (Australian Physiotherapy Association, 2020; Blacquiere et al., 2020; Canadian Chiropractic Guideline Initiative, 2020; Canadian Society for Exercise Physiology, 2020c; Dahlberg et al., 2016; Lear et al., 2021; Nyberg et al., 2017; Pineau et al., 2006; Reeves et al., 2021; Signal et al., 2020; Tomkins-Lane et al., 2015; Zhang, 2021). The chronic conditions represented in this review include lumbar spinal stenosis (Tomkins-Lane et al., 2015), arthritis (Australian Physiotherapy Association, 2020; Dahlberg et al., 2016), chronic obstructive pulmonary disease (Australian Physiotherapy Association, 2020; Lear et al., 2021; Nyberg et al., 2017), stroke (Australian Physiotherapy Association, 2020; Blacquiere et al., 2020; Signal et al., 2020), neurological disability (Signal et al., 2020), cardiovascular disease (Australian Physiotherapy Association, 2020; Lear et al., 2021), diabetes (Zhang, 2021), chronic kidney disease (Lear et al., 2021), and multimorbidity (Australian Physiotherapy Association, 2020; Lear et al., 2021).

Three studies (Canadian Chiropractic Guideline Initiative, 2020; Canadian Society for Exercise Physiology, 2020c; Pineau et al., 2006) provided generalized guidance applicable to various populations. Registered exercise physiologists, physiotherapists, occupational therapists, chiropractors, nurses, and multidisciplinary primary care teams, including dieticians, psychotherapists, and exercise specialists, were intended audiences for identified documents and/or implementing virtual interventions (Australian Physiotherapy Association, 2020; Blacquiere et al., 2020; Canadian Chiropractic Guideline Initiative, 2020; Canadian Society for Exercise Physiology, 2020c; Dahlberg et al., 2016; Lear et al., 2021; Nyberg et al., 2017; Pineau et al., 2006; Reeves et al., 2021; Signal et al., 2020; Tomkins-Lane et al., 2015; Zhang, 2021).

Formats for remote chronic disease management programs include video conferencing (Australian Physiotherapy Association, 2020; Blacquiere et al., 2020; Canadian Chiropractic Guideline Initiative, 2020; Canadian Society for Exercise Physiology, 2020c; Pineau et al., 2006; Signal et al., 2020), website platforms (Dahlberg et al., 2016; Lear et al., 2021; Nyberg et al., 2017; Signal et al., 2020; Tomkins-Lane et al., 2015), mobile application-based (Signal et al., 2020), and/or telephone-based (Australian Physiotherapy Association, 2020; Blacquiere et al., 2020; Signal et al., 2020). One document (Zhang, 2021) did not specify the format, referring generally to “telemedicine” as a delivery method (Zhang, 2021). Most studies/documents are from Canada (Blacquiere et al., 2020; Canadian Chiropractic Guideline Initiative, 2020; Canadian Society for Exercise Physiology, 2020c; Lear et al., 2021; Pineau et al., 2006; Tomkins-Lane et al., 2015), with the remainder from Sweden (Dahlberg et al., 2016; Nyberg et al., 2017), Australia (Australian Physiotherapy Association, 2020; Reeves et al., 2021), New Zealand (Signal et al., 2020), and China (Zhang, 2021). Table 2 presents the characteristics of the documents included.

**Table 2.** Characteristics of Included Documents

Author, Year	Country of origin	Reference type	Healthcare provider	Program Format	Chronic condition(s)
Dahlberg et al., 2016	Sweden	Journal article, pilot study	Physiotherapist Occupational Therapist	Website	Arthritis
Lear et al., 2021	Canada	Journal article, randomized control study	Nurse Dietician Exercise Specialist	Telephone Website	Diabetes Heart failure Ischemic heart disease Chronic kidney disease COPD
Nyberg et al., 2017	Sweden	Journal article, protocol paper	Primary care providers	Website	COPD
Reeves, 2021	Australia	Journal article, randomized control study	Registered Dietician^*^	Telephone	Cancer
Tomkins-Lane et al., 2015	Canada	Journal article, pilot study	Dietician Exercise Physiologist	Website	Lumbar spinal stenosis
Zhang, 2021	China	Journal article, consensus	Primary care providers	Telemedicine, unspecified	Diabetes
Pineau et al., 2006	Canada	Clinical guideline and technical standard	Rehabilitation care providers	Videoconferencing	Unspecified
Australian Physiotherapy Association, 2020	Australia	Guideline	Physiotherapists	Videoconferencing Telephone	Arthritis COPD
					Cancer Stroke Cardiovascular disease Cystic fibrosis Brain injury Multiple sclerosis Parkinson's
Blacquiere et al., 2020	Canada	Guideline	Physiotherapists Occupational therapists	Videoconferencing Telephone Email	Stroke
Canadian Chiropractic Guidelines Initiative, 2020	Canada	Guideline	Chiropractors	Videoconferencing Telephone	Unspecified
Canadian Society for Exercise Physiology, 2020	Canada	Guideline	Registered Exercise Physiologist	Videoconferencing Application	Unspecified
Signal et al. 2020	New Zealand	Journal article, Commentary	Physiotherapists	Videoconferencing	Stroke

All identified documents discussed issues regarding informed consent for remote chronic disease management programs (Australian Physiotherapy Association, 2020; Blacquiere et al., 2020; Canadian Chiropractic Guideline Initiative, 2020; Canadian Society for Exercise Physiology, 2020c; Dahlberg et al., 2016; Lear et al., 2021; Nyberg et al., 2017; Pineau et al., 2006; Reeves et al., 2021; Signal et al., 2020; Tomkins-Lane et al., 2015). All primary research studies included a statement about obtaining informed consent prior to starting the respective program, aligned with the respective research ethics boards (Dahlberg et al., 2016; Lear et al., 2021; Nyberg et al., 2017; Reeves et al., 2021; Tomkins-Lane et al., 2015). The other documents discussed consent in the context of reviewing and obtaining consent for remote care with participants (Blacquiere et al., 2020; Canadian Chiropractic Guideline Initiative, 2020), verification of a participant's identity (Blacquiere et al., 2020; Canadian Chiropractic Guideline Initiative, 2020), consent for the use of less secure program delivery formats such as Zoom, Skype, or FaceTime (Canadian Society for Exercise Physiology, 2020c), recording of remote sessions (Australian Physiotherapy Association, 2020), and intent to ensure privacy and confidentiality during the session (Australian Physiotherapy Association, 2020; Canadian Society for Exercise Physiology, 2020c; Pineau et al., 2006; Signal et al., 2020).

Two documents (Australian Physiotherapy Association, 2020; Pineau et al., 2006) discussed healthcare providers' prior training in remote care. One (Australian Physiotherapy Association, 2020) document described the importance of providers having familiarity with remote care, the ability to cope and manage the proposed technology, and understanding the strengths and weaknesses of remote care (Australian Physiotherapy Association, 2020). The other (Pineau et al., 2006) described the necessity of providers having adequate training prior to implementing remote programs to ensure the success of the program (Pineau et al., 2006). Both documents described the importance of remote care as equal to in-person care (Australian Physiotherapy Association, 2020; Pineau et al., 2006). One primary research article on the COPD-web (Nyberg et al., 2017) described training healthcare providers in the intervention over two sessions (Nyberg et al., 2017). The first session included information on the COPD-web, including processes and procedures (i.e., consent, outcome measures, and screening of participants), how content was supported by best practice guidelines, and how the intervention can facilitate participant self-management (Nyberg et al., 2017). The second training session discussed how the providers implemented the intervention in their daily practice and reinforced the study procedures (Nyberg et al., 2017).

Three documents (Canadian Society for Exercise Physiology, 2020c; Signal et al., 2020; Zhang, 2021) described pre-screening participants before remote chronic disease management for medical clearance for exercise (i.e., safe heart rate, blood pressure, medications, previous physical activity levels) (Canadian Society for Exercise Physiology, 2020c; Zhang, 2021) and safety concerns (i.e., fall risks, ability to get up off the floor, baseline mobility) (Signal et al., 2020). Medical clearance for moderate-to-high-intensity exercise was proposed to be in-person, including cardiovascular endurance, body composition, muscle strength, and flexibility (Zhang, 2021). Another document did not discuss a formal pre-screening process, but rather described the importance of identifying individuals for whom remote care may decrease delays in accessing care (Australian Physiotherapy Association, 2020).

One primary study and one document encouraged the use of motivational interviewing to improve engagement and provide education (Canadian Society for Exercise Physiology, 2020c; Reeves et al., 2021). Motivational interviews were conducted during phone calls with participants to encourage skill building, problem solving, and work toward sustained behavioural change (Reeves et al., 2021). Two other documents also identified coaching as a primary form of communication while providing education and reassurance to the participants (Australian Physiotherapy Association, 2020; Blacquiere et al., 2020).

The educational components described within primary research studies and other documents included disease-specific information (Australian Physiotherapy Association, 2020; Blacquiere et al., 2020; Canadian Chiropractic Guideline Initiative, 2020; Canadian Society for Exercise Physiology, 2020c; Dahlberg et al., 2016; Lear et al., 2021; Nyberg et al., 2017; Pineau et al., 2006; Reeves et al., 2021; Signal et al., 2020; Tomkins-Lane et al., 2015; Zhang, 2021), self-management (Australian Physiotherapy Association, 2020; Blacquiere et al., 2020; Canadian Chiropractic Guideline Initiative, 2020; Dahlberg et al., 2016; Nyberg et al., 2017; Signal et al., 2020; Zhang, 2021), lifestyle and behavioural factors (Australian Physiotherapy Association, 2020; Blacquiere et al., 2020; Canadian Chiropractic Guideline Initiative, 2020; Dahlberg et al., 2016; Reeves et al., 2021; Tomkins-Lane et al., 2015; Zhang, 2021), coping strategies (Blacquiere et al., 2020; Dahlberg et al., 2016; Nyberg et al., 2017; Zhang, 2021), and information on the program itself (Reeves et al., 2021). Two primary research studies also described the option to communicate and ask questions to healthcare providers using online portals (Nyberg et al., 2017) or chat functions (Dahlberg et al., 2016) as needed. Education was delivered in video format (Dahlberg et al., 2016; Tomkins-Lane et al., 2015), through website pages (Lear et al., 2021; Nyberg et al., 2017), workbooks (Reeves et al., 2021), links to external resources (Canadian Chiropractic Guideline Initiative, 2020; Signal et al., 2020), or through synchronous consultations with a healthcare provider (Australian Physiotherapy Association, 2020; Blacquiere et al., 2020; Canadian Chiropractic Guideline Initiative, 2020; Lear et al., 2021; Signal et al., 2020).

Various exercise recommendations have been described in all identified documents (Australian Physiotherapy Association, 2020; Blacquiere et al., 2020; Canadian Chiropractic Guideline Initiative, 2020; Canadian Society for Exercise Physiology, 2020c; Dahlberg et al., 2016; Lear et al., 2021; Nyberg et al., 2017; Pineau et al., 2006; Reeves et al., 2021; Signal et al., 2020; Tomkins-Lane et al., 2015; Zhang, 2021). Four documents described aerobic and/or resistance exercises (Canadian Chiropractic Guideline Initiative, 2020; Lear et al., 2021; Reeves et al., 2021; Zhang, 2021). Aerobic exercise was moderate to high intensity or as prescribed by the provider (Canadian Chiropractic Guideline Initiative, 2020; Lear et al., 2021; Reeves et al., 2021; Zhang, 2021). One primary research article (Reeves et al., 2021) and one document (Zhang, 2021) specified 150 (Zhang, 2021) and 210 (Reeves et al., 2021) minutes of aerobic activity per week respectively. Common aerobic exercises prescribed included walking, biking, swimming (Reeves et al., 2021), or other activities identified by participants that aligned with their chronic condition(s) (Canadian Chiropractic Guideline Initiative, 2020; Lear et al., 2021; Reeves et al., 2021; Zhang, 2021). Resistance exercises were described in three primary research studies (Dahlberg et al., 2016; Lear et al., 2021; Reeves et al., 2021) and included four neuromuscular strengthening exercises for osteoarthritis (Dahlberg et al., 2016), two-to-three resistance exercises per week for the living well after breast cancer trial (Reeves et al., 2021), and on an “as needed” basis in the internet chronic disease management program (Lear et al., 2021). Other documents included strength exercises (Blacquiere et al., 2020; Canadian Chiropractic Guideline Initiative, 2020), and one document detailed resistance training for two-to-three days per week at moderate intensity, targeting all major muscle groups (Zhang, 2021).

### Primary Research Articles

Three primary research studies discussed theories on which interventions were developed (Dahlberg et al., 2016; Reeves et al., 2021; Tomkins-Lane et al., 2015). Dahlberg et al. 2016 and Tomkins-Lane et al 2015 described the use of behavioural change strategies and Reeves et al. 2021 described the use of social cognitive theory. Of the five primary research studies, four provided descriptions of action planning (Lear et al., 2021), goal setting (Lear et al., 2021; Reeves et al., 2021; Tomkins-Lane et al., 2015), self-monitoring (Lear et al., 2021; Reeves et al., 2021; Tomkins-Lane et al., 2015), problem-solving (Reeves et al., 2021), and social support (Nyberg et al., 2017; Reeves et al., 2021; Tomkins-Lane et al., 2015). Two studies used group discussion boards for participants to communicate with one another as a means of social support (Nyberg et al., 2017; Tomkins-Lane et al., 2015) and one emphasized the importance of social support within the intervention (Reeves et al., 2021).

Four primary research studies were web-based interventions (Dahlberg et al., 2016; Lear et al., 2021; Nyberg et al., 2017; Tomkins-Lane et al., 2015). Three websites provided individualized packages to the participants using email (Dahlberg et al., 2016) or a unique log-in to the website forum (Lear et al., 2021; Tomkins-Lane et al., 2015). Participants were either oriented to the website by a healthcare provider via telephone in one intervention (Lear et al., 2021) or navigated through the website on their own (Nyberg et al., 2017; Tomkins-Lane et al., 2015). One primary research article used phone calls and text messages (Reeves et al., 2021).

Education was provided to the participants in a variety of formats, including structured and informal interactions (Dahlberg et al., 2016; Lear et al., 2021; Nyberg et al., 2017; Reeves et al., 2021; Tomkins-Lane et al., 2015). Structured education included interactive 10-minute sessions (Tomkins-Lane et al., 2015), two- to five-minute video lectures with an accompanying quiz (Dahlberg et al., 2016), workbooks (Reeves et al., 2021), sessions with a healthcare provider via telephone (Reeves et al., 2021) and dedicated webpages (Lear et al., 2021; Nyberg et al., 2017). Education was also provided informally using follow-up visits with a healthcare provider (Lear et al., 2021; Reeves et al., 2021) or communicating with physiotherapists or content experts through asynchronous portals (Dahlberg et al., 2016; Nyberg et al., 2017).

Pedometer-based exercises have been described in three primary research studies (Nyberg et al., 2017; Reeves et al., 2021; Tomkins-Lane et al., 2015). Participants were provided with a pedometer with instructions on how to use it in two studies (Nyberg et al., 2017; Tomkins-Lane et al., 2015). Two studies described having space for participants to record their steps (Nyberg et al., 2017; Tomkins-Lane et al., 2015), and one described the option for personalized feedback on progress (Nyberg et al., 2017). One article described setting a goal of 10,000 steps for participants (Reeves et al., 2021), while another provided personalized step-goal tips on how to achieve the step goal for the day (Tomkins-Lane et al., 2015). Other exercise programs included home-based aerobic and resistance training, as described previously (Dahlberg et al., 2016; Lear et al., 2021; Reeves et al., 2021). There were several supports for the activity described, including videos that outline how to complete exercises (Dahlberg et al., 2016), information on physical activity and training techniques via websites (Nyberg et al., 2017), and walking maps of communities (Tomkins-Lane et al., 2015).

### Other Documents

Establishing emergency procedures in case of an adverse event or participant deterioration was discussed in five documents (Australian Physiotherapy Association, 2020; Blacquiere et al., 2020; Canadian Chiropractic Guideline Initiative, 2020; Pineau et al., 2006; Signal et al., 2020). Three documents identified the need for safety plans (Australian Physiotherapy Association, 2020; Blacquiere et al., 2020; Signal et al., 2020). In one document, this included identifying if someone else is in the home (while maintaining privacy), (Canadian Society for Exercise Physiology, 2020c) and in others, proactively identifying potential hazards before starting the session (Australian Physiotherapy Association, 2020; Canadian Society for Exercise Physiology, 2020c; Signal et al., 2020). Another document described obtaining participants' emergency contact information (name and phone number) and the importance of providers being able to recognize when participants should seek in-person or emergency care (Canadian Chiropractic Guideline Initiative, 2020).

Education was tailored to individual participants in three documents (Australian Physiotherapy Association, 2020; Signal et al., 2020; Zhang, 2021). One document described specific time points that education should be provided to participants, including at the time of diagnosis of the chronic condition, during annual assessments, when self-management is impacted, during exacerbation of symptoms, and during transitional care (Zhang, 2021). Other documents did not specify the time period for delivery of education (Australian Physiotherapy Association, 2020; Blacquiere et al., 2020; Canadian Chiropractic Guideline Initiative, 2020; Canadian Society for Exercise Physiology, 2020c; Pineau et al., 2006; Signal et al., 2020). Education was delivered via telephone, videoconferencing (Australian Physiotherapy Association, 2020; Blacquiere et al., 2020; Canadian Chiropractic Guideline Initiative, 2020; Canadian Society for Exercise Physiology, 2020c), or email (Signal et al., 2020). Telephone and/or videoconferencing education was described as coaching and included condition-specific information, self-management support, physical activity advice, and status updates on participant progress (Australian Physiotherapy Association, 2020; Blacquiere et al., 2020; Canadian Chiropractic Guideline Initiative, 2020; Canadian Society for Exercise Physiology, 2020c). One source described emailing participants with links to additional resources that supported problem-solving, empowerment, and capacity building for self-management (Signal et al., 2020). Another resource described directing participants to other external resources to complement education, such as YouTube or mindfulness applications; however, it was not clear how these applications or specific videos were chosen (Canadian Chiropractic Guideline Initiative, 2020).

The set-up required for exercise was discussed in four documents (Australian Physiotherapy Association, 2020; Blacquiere et al., 2020; Canadian Society for Exercise Physiology, 2020c; Pineau et al., 2006), and one document provided detailed guidance on the set-up for the remote exercise (Pineau et al., 2006). Space set-up requirements, such as the size of the room (10'x15'), contrasting background colour, and adequate lighting, were described in one document (Pineau et al., 2006). The technology requirements were described, including audio equipment (Australian Physiotherapy Association, 2020; Blacquiere et al., 2020; Canadian Society for Exercise Physiology, 2020c; Pineau et al., 2006), camera(s) (Australian Physiotherapy Association, 2020; Blacquiere et al., 2020; Canadian Society for Exercise Physiology, 2020c; Pineau et al., 2006), and a monitor (Pineau et al., 2006). One document also described the orientation of individuals toward safe participation by reviewing appropriate clothing and footwear and obtaining the necessary exercise equipment (Signal et al., 2020).

Exercise prescription was detailed in one document (Zhang, 2021) and included 150 minutes of moderate-intensity aerobic exercise per week over three days, starting with a warm-up and ending with a cool-down and relaxation exercises (Zhang, 2021). Resistance training was prescribed two-to-three times per week on non-consecutive days at moderate intensity, covering all muscle groups (Zhang, 2021). The authors also prescribed exercise one-to-three-hours after a meal (Zhang, 2021). Two other documents specified the inclusion of aerobic exercise, resistance training, and flexibility within exercise programs (Blacquiere et al., 2020; Canadian Chiropractic Guideline Initiative, 2020), and one clarified that exercise parameters were clearly communicated to the participants (Blacquiere et al., 2020). Several other documents have described the process of teaching exercises, starting with a visual demonstration by the provider, followed by an attempt by the participant with feedback (Australian Physiotherapy Association, 2020; Canadian Chiropractic Guideline Initiative, 2020; Signal et al., 2020). One document described breaking down each exercise into smaller movements for teaching purposes and progressing exercises one at a time, clarifying the starting and ending positions (Signal et al., 2020). Exercise sessions were supplemented with written or picture references (Blacquiere et al., 2020; Canadian Chiropractic Guideline Initiative, 2020; Signal et al., 2020) or with a recommended application (Australian Physiotherapy Association, 2020; Blacquiere et al., 2020; Canadian Society for Exercise Physiology, 2020c). One document described following up with participants after exercise sessions with key-take-home messages to reinforce content (Signal et al., 2020).

**Figure 2.**
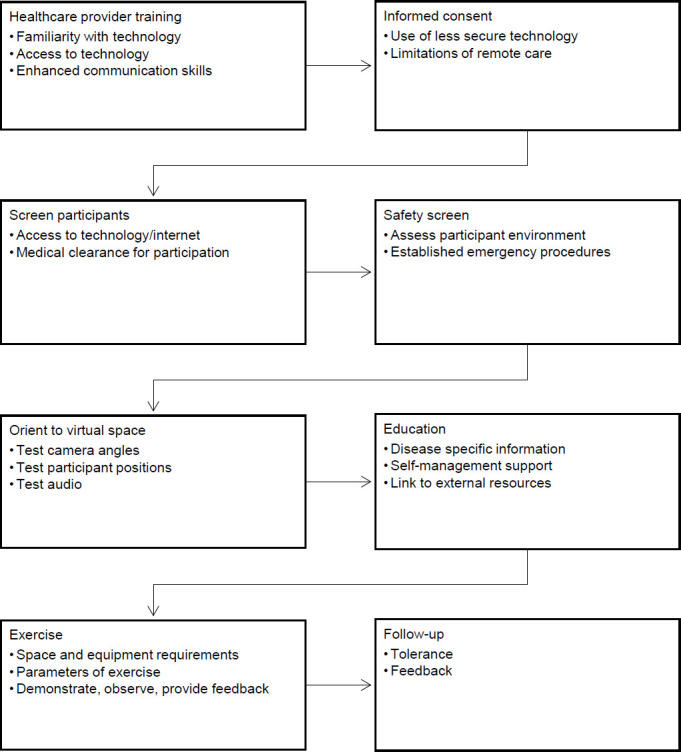
Visual Representation of Overall Scoping Review Findings Regarding the Processes and Procedures for Remote Chronic Disease Management

Communication is described in four documents (Blacquiere et al., 2020; Canadian Chiropractic Guideline Initiative, 2020; Pineau et al., 2006; Signal et al., 2020). Effective communication has been described as integral to developing and maintaining a therapeutic relationship (Blacquiere et al., 2020; Canadian Chiropractic Guideline Initiative, 2020; Pineau et al., 2006; Signal et al., 2020). Recommended strategies for building a remote therapeutic relationship included demonstrating empathy (Pineau et al., 2006; Signal et al., 2020), providing validation (Blacquiere et al., 2020), acknowledging participants' experiences, emotions, and perspectives (Signal et al., 2020), and allowing for pro-social talk during sessions (Signal et al., 2020). One document acknowledged that participants were welcoming providers into their spaces at the start of the sessions (Blacquiere et al., 2020). One document also described the need for improved cueing of participants during the exercise sessions (Blacquiere et al., 2020). Nonverbal communication and active listening strategies were also described, including the use of gestures, facial expressions, and nodding (Blacquiere et al., 2020; Canadian Chiropractic Guideline Initiative, 2020; Signal et al., 2020). Figure 2 provides a visual representation of the study findings, summarizing the process of remote chronic disease management.

## Discussion

This review identified 12 documents that provided guidance on remote chronic disease management that addressed both exercise and education. The identified documents were heterogeneous, including a variety of journal articles such as protocol studies (Nyberg et al., 2017), pilot studies (Dahlberg et al., 2016; Tomkins-Lane et al., 2015), randomized controlled studies (Lear et al., 2021; Reeves et al., 2021), consensus article (Zhang, 2021), and commentary (Signal et al., 2020), in addition to diverse presentations of grey literature guideline documents (Australian Physiotherapy Association, 2020; Blacquiere et al., 2020; Canadian Chiropractic Guideline Initiative, 2020; Canadian Society for Exercise Physiology, 2020c; Pineau et al., 2006). There were few primary research studies identified, and those identified focused on the effectiveness of the intervention rather than a larger discussion on the implementation of the interventions (Dahlberg et al., 2016; Lear et al., 2021; Nyberg et al., 2017; Reeves et al., 2021; Tomkins-Lane et al., 2015). While the focus on effectiveness is unsurprising, as it aligned with the stated research objectives of the respective studies, it highlights an implementation science gap within the literature on remote care. Relatively few other documents were identified, and it is surprising that many regulatory bodies and professional organizations provided limited guidance on implementing remote education and exercises.

Several studies within this review utilized pedometer-based walking programs as exercise components (Nyberg et al., 2017; Reeves et al., 2021; Tomkins-Lane et al., 2015). Walking is an accessible, low-risk activity with substantial health benefits for individuals with chronic conditions (Lee & Buchner, 2008). However, walking alone does not fully align with the recently published 24-hour movement guidelines from the Canadian Society for Exercise Physiology (CSEP) (Canadian Society for Exercise Physiology, 2020b, 2020a), which advocates for the inclusion of aerobic and resistance training of all major muscle groups, flexibility, and range of motion exercises (Canadian Society for Exercise Physiology, 2020a, 2020b). Other documents describe exercises with progressions and regressions to accommodate various abilities (Dahlberg et al., 2016; Signal et al., 2020). Having resources that outline variations of exercises helps manage symptom fluctuations that are expected when living with a chronic disease (Wagner et al., 2022).

All documents within this review targeted one-on-one healthcare provider-participant interactions, a missed opportunity for creating remote peer support communities to support self-management. The eHealth Enhanced Chronic Care Model (eCCM), derived from the widely accepted care model for managing chronic diseases, identifies that virtual communities can provide support to participants and result in more engaged, informed, and confident individuals who can better manage their chronic conditions (Gee et al., 2015). The role of peer support in diabetes management was examined qualitatively, with participants indicating favorable outcomes, including better engagement, information provision, self-monitoring, action planning, and goal prioritization because of group sessions (Borek et al., 2019). However, given the lack of guidance on how to implement group remote chronic disease management, more evidence is needed to support clinicians in implementing group programs online.

Several documents included in this review identified the importance of enhanced communication abilities among clinicians providing remote care, with two specifying motivational interviewing as an effective method. Motivational interviewing is a technique that seeks to resolve the uncertainty often experienced by individuals seeking change by focusing on communication with participants regarding the desired change (Purath et al., 2014). Focusing on the desired change during conversations can help participants understand their personal motivations for wanting to change and develop more effective goals and action plans (Purath et al., 2014). In a remote program, motivational interviewing can help identify barriers, beliefs, motivations, and emotions (Purath et al., 2014) regarding participation virtually and provide space for collaboration to develop action plans that are both valued and achievable by the participant. Motivational interviewing is a skill that becomes more effective with formal training (Purath et al., 2014), and clinicians aiming to implement virtual care would benefit from seeking training opportunities.

Although remote chronic disease management programs may help increase access to care for some individuals, it is important to acknowledge that they are not without limitations. Videoconferencing requires resources that are inaccessible in all contexts. Adequate Internet services are not available in ‘digitally isolated regions’, as in certain rural and remote communities, and further perpetuate health disparities already experienced in these locations (Hirko et al., 2020). The cost of devices (i.e., tablets, laptops, and smartphones) necessary to participate in virtual programs is high and may make virtual programs inaccessible, as chronic disease disproportionately impacts those with lower socioeconomic status (Public Health Agency of Canada, 2020). For remote programs to be impactful for communities without specialized clinicians, significant work is needed to improve access to reliable internet (Hirko et al., 2020), and resources to access the technology are necessary.

A policy review of the legislation may be beneficial for understanding the legalities of providing remote care from a privacy and confidentiality perspective as well as funding and billing perspectives. A more detailed review of the Canadian regulatory bodies' position on remote care may also allow for clarification of the current roles and responsibilities, while highlighting any changes that may need to be advocated to facilitate better access to remote chronic disease management programs. For example, healthcare providers must be in the same province or territory at the time of care in Canada to provide care virtually (Government of Canada, 2019); however, with limited access to healthcare providers in rural and remote areas, this may create unnecessary barriers to qualified practitioners.

### Strengths

This review has strengths in the methodology used to identify, map, and synthesize available evidence. Numerous databases were searched in addition to grey literature sources (targeted websites, Google, CADTH grey literature database, and Canadian Politics Collection). The systematic search was completed in July 2021 and was updated in May 2022. The dates of the review coincided with the move to remote programs in response to the COVID-19 pandemic. Two reviewers were used at each stage of the scoping review in alignment with best practices.

### Limitations

Due to our inclusion criteria, studies that provided guidance on virtual education or virtual exercise only were excluded. However, given the importance of both education and exercise, authors chose to focus only on documents that included both components. Existing reviews exist on remote chronic disease education (Warsi et al., n.d.) and exercises (Brown et al., 2022). Reviews of education alone have demonstrated that virtual education and social support produce similar outcomes to in-person education (i.e., improved mental health, accessibility to care, development of health knowledge, and skills) (Banbury et al., 2018; Warsi et al., n.d.). Similarly, reviews on exercise alone are also available that demonstrate the feasibility of virtual or remote exercise with improvements in exercise capacity and quality of life (Brown et al., 2022). Primary research studies comparing remote programs to in-person (i.e., the comparison group was in-person) were excluded from the review, and these studies might have provided information on how to implement remote programs.

Only licensed professionals were included in this review, which may have limited our results. Some researchers may have trained research assistants or utilized personal trainers and other unregulated providers to deliver the remote program. Only documents published in English were included because of language and resource limitations of the research team.

## Conclusions

Remote chronic disease management programs, including exercise and education, have the potential to improve access to care. However, there is limited guidance and research available on how to implement a remote chronic disease management program that incorporates both education and exercise and is delivered by a registered health care professional. Current guidelines focus on one-on-one care, which may limit the impact of virtual group programmes and communities on chronic disease management.

## Availability of Data, Code, and Other Materials

Data extraction templates and data available upon request from corresponding author.

## References

[B1] Allen, K. D., Oddone, E. Z., Coffman, C. J., Datta, S. K., Juntilla, K. A., Lindquist, J. H., Walker, T. A., Weinberger, M., & Bosworth, H. B. (2010). Telephone-based self-management of Osteoarthritis. Annals of Internal Medicine, 153, 570–579. 21041576 10.7326/0003-4819-153-9-201011020-00006

[B2] Antypas, K., & Wangberg, S. C. (2014). An internet- and mobile-based tailored intervention to enhance maintenance of physical activity after cardiac rehabilitation: Short-term results of a randomized controlled trial. Journal of Medical Internet Research, 16(3), 1–18. 10.2196/jmir.3132 PMC3967125 24618349

[B3] Australian Physiotherapy Association. (2020). Telehealth Guidelines Response to COVID-19.

[B4] Banbury, A., Nancarrow, S., Dart, J., Gray, L., & Parkinson, L. (2018). Telehealth interventions delivering home-based support group videoconferencing: Systematic review. In Journal of Medical Internet Research (Vol. 20, Issue 2). JMIR Publications Inc. 10.2196/jmir.8090 PMC5816261 29396387

[B5] Bannuru, R. R., Osani, M. C., Vaysbrot, E. E., Arden, N. K., Bennell, K., Bierma-Zeinstra, S. M. A., Kraus, V. B., Lohmander, L. S., Abbott, J. H., Bhandari, M., Blanco, F. J., Espinosa, R., Haugen, I. K., Lin, J., Mandl, L. A., Moilanen, E., Nakamura, N., Snyder-Mackler, L., Trojian, T., … McAlindon, T. E. (2019). OARSI guidelines for the non-surgical management of knee, hip, and polyarticular osteoarthritis. Osteoarthritis and Cartilage, 27(11), 1578–1589. 10.1016/j.joca.2019.06.011 31278997

[B6] Blacquiere, D., Gubitz, G., YX Yu, A., Wein, T., McGuff, R., Pollard, J., Smith, E., Mountain, A., & Lindsay, M. P. (2020). Canadian Stroke Best Practice Recommendations, 7th Edition: Virtual Care (Telestroke) Implementation Toolkit.

[B7] Bodenheimer, T., Lorig, K., Holman, H., & Grumbach, K. (2002). Patient Self-management of Chronic Disease in Primary Care. INNOVATIONS IN PRIMARY CARE. https://jamanetwork.com/10.1001/jama.288.19.2469 12435261

[B8] Borek, A. J., Abraham, C., Greaves, C. J., Tarrant, M., Garner, N., & Pascale, M. (2019). ‘We're all in the same boat’: A qualitative study on how groups work in a diabetes prevention and management programme. British Journal of Health Psychology, 24(4), 787–805. 10.1111/bjhp.12379 31273908

[B9] Brown, R. C. C., Coombes, J. S., Jungbluth Rodriguez, K., Hickman, I. J., & Keating, S. E. (2022). Effectiveness of exercise via telehealth for chronic disease: a systematic review and meta-analysis of exercise interventions delivered via videoconferencing. In British Journal of Sports Medicine (Vol. 56, Issue 18, pp. 1042–1052). BMJ Publishing Group. 10.1136/bjsports-2021-105118 35715175

[B10] Canadian Chiropractic Guideline Initiative. (2020). Provide education and self-management strategies: Clinician Summary-Telehealth.

[B11] Canadian Society for Exercise Physiology. (2020a). Canadian 24-hour movement guidelines for Adults 18–64.

[B12] Canadian Society for Exercise Physiology. (2020b). Canadian 24-hour movement guidelines for Adults 65+. https://csep.ca/guidelines.

[B13] Canadian Society for Exercise Physiology. (2020c). Telehealth Training and Counselling Guidelines for CSEP Certified Members CSEP.

[B14] Dahlberg, L. E., Grahn, D., Dahlberg, J. E., & Thorstensson, C. A. (2016). A Web-Based Platform for Patients with Osteoarthritis of the Hip and Knee: A Pilot Study. JMIR Research Protocols, 5(2), e115. 10.2196/resprot.5665 27261271 PMC4912680

[B15] Dal Bello-Haas, V. P. M., O'Connell, M. E., Morgan, D. G., & Crossley, M. (2014). Lessons learned: Feasibility and acceptability of a telehealth-delivered exercise intervention for rural-dwelling individuals with dementia and their caregivers. Rural and Remote Health, 14(3), 1–11. 25081991

[B16] Fortin, M., Chouinard, M. C., Bouhali, T., Dubois, M. F., Gagnon, C., & Bélanger, M. (2013). Evaluating the integration of chronic disease prevention and management services into primary health care. BMC Health Services Research, 13(1). 10.1186/1472-6963-13-132 PMC3637600 23565674

[B17] Fortin, M., Chouinard, M.-C., Dubois, M.-F., Belanger, M., Almirall, J., Bouhali, T., & Sasseville, M. (2016). Integration of chronic disease prevention and management services into primary care: a pragmatic randomized controlled trial (PR1MaC). CMAJ Open, 4(4), E588–E598. 10.9778/cmajo.20160031 PMC5173473 28018871

[B18] Gee, P. M., Greenwood, D. A., Paterniti, D. A., Ward, D., & Miller, L. M. S. (2015). The eHealth enhanced chronic care model: A theory derivation approach. Journal of Medical Internet Research, 17(4), e86. 10.2196/jmir.4067 25842005 PMC4398883

[B19] Government of Canada. (2019, September 17). Canada's Health Care System.

[B20] Hajat, C., & Stein, E. (2018). The global burden of multiple chronic conditions: A narrative review. In Preventive Medicine Reports (Vol. 12, pp. 284–293). Elsevier Inc. 10.1016/j.pmedr.2018.10.008 30406006 PMC6214883

[B21] Harkey, L. C., Jung, S. M., Newton, E. R., & Patterson, A. (2020). Patient satisfaction with telehealth in rural settings: A systematic review. In International Journal of Telerehabilitation, 12(2), 53–64. 10.5195/ijt.2020.6303 33520095 PMC7757651

[B22] Hevey, D., Wilson O'Raghallaigh, J., O'Doherty, V., & Lonergan, K. (2018). Pre-post effectiveness evaluation of Chronic Disease Self-Management Program (CDSMP) participation on health, well-being and health service utilization. Chronic Illness. 10.1177/1742395318792063 30089405

[B23] Hirko, K. A., Kerver, J. M., Ford, S., Szafranski, C., Beckett, J., Kitchen, C., & Wendling, A. L. (2020). Telehealth in response to the COVID-19 pandemic: Implications for rural health disparities. Journal of the American Medical Informatics Association, 27(11), 1816–1818. 10.1093/jamia/ocaa156 32589735 PMC7337797

[B24] Hwang, W., Weller, W., Ireys, H., & Anderson, G. (2001). Out-of-pocket medical spending for care of chronic conditions. Health Affairs, 20(6), 267–278. 10.1377/hlthaff.20.6.267 11816667

[B25] Katz, I. J., Pirabhahar, S., Williamson, P., Raghunath, V., Brennan, F., Sullivan, A. O., Youssef, G., Lane, C., Jacobson, G., Feldman, P., & Kelly, J. (2018). iConnect CKD – virtual medical consulting: A web-based chronic kidney disease, hypertension and diabetes integrated care program. 646–652. 10.1111/nep.13070 28474361

[B26] Lear, S. A., Norena, M., Banner, D., Whitehurst, D. G. T., Gill, S., Burns, J., Kandola, D. K., Johnston, S., Horvat, D., Vincent, K., Levin, A., Kaan, A., van Spall, H. G. C., & Singer, J. (2021). Assessment of an interactive digital health-based self-management program to reduce hospitalizations among patients with multiple chronic diseases: A randomized clinical trial. JAMA Network Open, 4(12). 10.1001/jamanetworkopen.2021.40591 PMC12243620 34962560

[B27] Lee, I. M., & Buchner, D. M. (2008). The importance of walking to public health. Medicine and Science in Sports and Exercise, 40(7 SUPPL. 1). 10.1249/MSS.0b013e31817c65d0 18562968

[B28] Lewis, E., Samperi, S., & Boyd-Skinner, C. (2017). Telephone follow-up calls for older patients after hospital discharge. Age and Ageing, 46(4), 544–546. 10.1093/ageing/afw251 28104599

[B29] Moore, S. M., Schiffman, R., Waldrop-valverde, D., Redeker, N. S., Mccloskey, D. J., Kim, M. T., Heitkemper, M. M., Guthrie, B. J., Dorsey, S. G., Docherty, S. L., Barton, D., Bailey, D. E., Austin, J., & Grady, P. (2016). Recommendations of common data elements to advance the science of self-management of chronic conditions. 48(5), 437–447. 10.1111/jnu.12233 PMC5490657 27486851

[B30] Nyberg, A., Wadell, K., Lindgren, H., & Tistad, M. (2017). Internet-based support for self-management strategies for people with COPD-protocol for a controlled pragmatic pilot trial of effectiveness and a process evaluation in primary healthcare. BMJ Open, 7(7). 10.1136/bmjopen-2017-016851 PMC5642786 28765136

[B31] Peters, M. D. J., Godfrey, C., McInerney, P., Munn, Z., Tricco, A. C., & Khalil, H. (2020). Scoping Reviews. In E. Aromataris & Z. Munn (Eds.), JBI Manual for evidence synthesis (2020 versi). JBI. 10.46658/JBIMES-20-12

[B32] Pineau, G., Moqadem, K., St-Hilaire, C., Levac, E., & Hamel, B. (2006). Telehealth: Clinical Guidelines and Technical Standards for Telerehabilitation.

[B33] Portnoy, J., Waller, M., & Elliott, T. (2020). Telemedicine in the Era of COVID-19. Journal of Allergy and Clinical Immunology: In Practice, 8(5), 1489–1491. 10.1016/j.jaip.2020.03.008 32220575 PMC7104202

[B34] Public Health Agency of Canada. (2020). Aging and chronic diseases: A profile of Canadian Seniors.

[B35] Purath, J., Keck, A., & Fitzgerald, C. E. (2014). Motivational interviewing for older adults in primary care: A systematic review. Geriatric Nursing, 35(3), 219–224. 10.1016/j.gerinurse.2014.02.002 24656051

[B36] Reeves, M. M., Terranova, C. O., Winkler, E. A. H., McCarthy, N., Hickman, I. J., Ware, R. S., Lawler, S. P., Eakin, E. G., & Demark-Wahnefried, W. (2021). Effect of a remotely delivered weight loss intervention in early-stage breast cancer: Randomized controlled trial. Nutrients, 13(11). 10.3390/nu13114091 PMC8622393 34836345

[B37] Seron, P., Oliveros, M. J., Gutierrez-Arias, R., Fuentes-Aspe, R., Torres-Castro, R. C., Merino-Osorio, C., Nahuelhual, P., Inostroza, J., Jalil, Y., Solano, R., Marzuca-Nassr, G. N., Aguilera-Eguía, R., Lavados-Romo, P., Soto-Rodríguez, F. J., Sabelle, C., Villarroel-Silva, G., Gomolán, P., Huaiquilaf, S., & Sanchez, P. (2021). Effectiveness of telerehabilitation in physical therapy: A rapid overview. Physical Therapy, 101(6). 10.1093/ptj/pzab053 PMC7928601 33561280

[B38] Signal, N., Martin, T., Leys, A., Maloney, R., & Bright, F. (2020). Implementation of telerehabilitation in response to COVID-19: Lessons learnt from neurorehabilitation clinical practice and education. New Zealand Journal of Physiotherapy, 48(3), 117–126. 10.15619/NZJP/48.3.03

[B39] Thornton, J. S., Frémont, P., Khan, K., Poirier, P., Fowles, J., Wells, G. D., & Frankovich, R. J. (2016). Physical activity prescription: A critical opportunity to address a modifiable risk factor for the prevention and management of chronic disease: A position statement by the Canadian Academy of Sport and Exercise Medicine. table 1, 1–6. 10.1136/bjsports-2016-096291 27359294

[B40] Tomkins-Lane, C. C., Lafave, L. M. Z., Parnell, J. A., Rempel, J., Moriartey, S., Andreas, Y., Wilson, P. M., Hepler, C., Ray, H. A., & Hu, R. (2015). The spinal stenosis pedometer and nutrition lifestyle intervention (SSPANLI): Development and pilot. Spine Journal, 15(4), 577–586. 10.1016/j.spinee.2014.10.015 25452012

[B41] Tricco, A. C., Lillie, E., Zarin, W., O'Brien, K. K., Colquhoun, H., Levac, D., Moher, D., Peters, M. D. J., Horsley, T., Weeks, L., Hempel, S., Akl, E. A., Chang, C., McGowan, J., Stewart, L., Hartling, L., Aldcroft, A., Wilson, M. G., Garritty, C., … Straus, S. E. (2018). PRISMA extension for scoping reviews (PRISMA-ScR): Checklist and explanation. Annals of Internal Medicine, 169(7), 467–473. 10.7326/M18-0850 30178033

[B42] Van Damme J, Dal Bello-Haas V. Guiding documents for healthcare providers engaging with virtual chronic disease management programs: A scoping review protocol. Open Science Framework. Published online June 2021.

[B43] Wagner, E. H., Austin, B. T., Davis, C., Hindmarsh, M., Schaefer, J., & Bonomi, A. (2022). Improving chronic illness care: Translating evidence into action. Health Aff (Millwood), 20(6), 64–78. 10.1377/hlthaff.20.6.64 11816692

[B44] Warsi, A., Wang, P. S., Lavalley, M. P., Avorn, J., & Solomon, D. H. (n.d.). Self-management education programs in chronic diseases: A systematic review and methodological critique of the literature. https://jamanetwork.com/10.1001/archinte.164.15.1641 15302634

[B45] Williams, R. (2012). Telemedicine in Ontario: Fact not fiction. Overview of telemedicine in Ontario. 1–4.

[B46] Zhang, B. (2021). Expert consensus on telemedicine management of diabetes (2020 edition). International Journal of Endocrinology, 2021. 10.1155/2021/6643491 PMC8016587 33833798

